# Root induced changes of effective 1D hydraulic properties in a soil column

**DOI:** 10.1007/s11104-014-2121-x

**Published:** 2014-04-28

**Authors:** P. Scholl, D. Leitner, G. Kammerer, W. Loiskandl, H.-P. Kaul, G. Bodner

**Affiliations:** 1 Department of Crop Sciences, Division of Agronomy, University of Natural Resources and Life Sciences, Konrad-Lorenz-Strasse 24, 3430 Tulln, Austria; 2 Institute of Hydraulics and Rural Water Management, University of Natural Resources and Life Sciences, Muthgasse 18, 1190 Vienna, Austria; 3 Computational Science Center, University of Vienna, Oskar Morgenstern-Platz 1, 1090 Vienna, Austria

**Keywords:** Pore size distribution, Plant roots, Column experiment, Pore evolution model, Cover crops

## Abstract

**Aims:**

Roots are essential drivers of soil structure and pore formation. This study aimed at quantifying root induced changes of the pore size distribution (PSD). The focus was on the extent of clogging vs. formation of pores during active root growth.

**Methods:**

Parameters of Kosugi’s lognormal PSD model were determined by inverse estimation in a column experiment with two cover crops (mustard, rye) and an unplanted control. Pore dynamics were described using a convection–dispersion like pore evolution model.

**Results:**

Rooted treatments showed a wider range of pore radii with increasing volumes of large macropores >500 μm and micropores <2.5 μm, while fine macropores, mesopores and larger micropores decreased. The non-rooted control showed narrowing of the PSD and reduced porosity over all radius classes. The pore evolution model accurately described root induced changes, while structure degradation in the non-rooted control was not captured properly. Our study demonstrated significant short term root effects with heterogenization of the pore system as dominant process of root induced structure formation.

**Conclusions:**

Pore clogging is suggested as a partial cause for reduced pore volume. The important change in micro- and large macropores however indicates that multiple mechanic and biochemical processes are involved in root-pore interactions.

## Introduction

Soil hydraulic properties are the common result of particle size distribution (texture) and aggregation (structure). Beside texture, soil structure is the main property to shape the fundamental relations for soil water flow, i.e. retention and hydraulic conductivity, in the saturated and near-saturated range (Cresswell et al. 1992). Among the various driving factors of soil structural porosity, vegetation is playing a dominant role. Roots are a key element in plant related effects on soil structure and soil hydrology (Gregory 2006; Bengough 2012; Logsdon 2013). Recently, Carminati et al. (2010) and Moradi et al. (2011) demonstrated that contrary to the usual assumption of higher depletion in vicinity of roots (Gardner 1960), soil water content is higher in rhizosphere soil compared to bulk soil over a wide range of pressure heads, indicating a significant change of the water retention curve in vicinity of plant roots.

Several pathways of root influence on soil hydraulic properties have been described. Temporal pore clogging occurs due to roots growing into pre-existing pores (e.g. Gish and Jury 1983; Morgan et al. 1995). Scanlan (2009) suggested that root in-growth results in the division of larger into smaller pores. Micro-fissures and cracks are structural pores formed in the root zone of transpiring plants by more intense wetting-drying (e.g. Dexter 1987; Mitchell et al. 1995; Young 1998; Whalley et al. 2005). These effects are depending on the lifespan of roots. Meek et al. (1990) and Murphy et al. (1993) measured reduced infiltration rates as long as plants are actively growing and their roots block pore channels. After root decay, bio-macropores and root-induced micropores are formed (Cresswell and Kirkegaard 1995; Mitchell et al. 1995; Wuest 2001; Horn and Smucker 2005; Ghestem et al. 2011). These pores have high connectivity (Pagliai and De Nobili 1993; Whalley et al. 2005), thereby facilitating water transport through the soil (Gish and Jury 1983; Murphy et al. 1993; Suwardji and Eberbach 1998). As a result, e.g. Disparte (1987) measured an increase of infiltration with higher rooting density after decomposition of roots. Gyssels et al. (2005) confirmed this finding by establishing a direct relation between root density and reduction of soil erosion.

The extent of root induced changes of soil hydraulic properties is influenced by both soil and root characteristics. Scanlan (2009) did not find any root effect on soil hydraulic properties in a column experiment using a sandy substrate. We suppose that changes of pore properties depend strongly on (i) the extent of existing growth paths for root penetration (Feeney et al. 2006) and (ii) the relation between root volume and pore volume (Bengough 2012). Yunusa and Newton (2003) reported differences among species in their effects on soil hydraulic properties. Perennials and woody plants substantially changed flow behavior while annual crops had hardly any influence. Among annual plants they suggested root diameter as main trait for effectively priming soil hydraulic properties. Higher strength of coarse roots allows more effective shift of soil particles and lower tendency of root buckling under mechanical stress (Clark et al. 2003). Coarser root axes exert higher radial pressures among soil penetration, thereby enlarging existing pores while increasing density of adjacent rhizosphere soil (Dexter 1987; Archer et al. 2002; Kirby and Bengough 2002; Whalley et al. 2004).

A major challenge for understanding root effects on soil hydraulic properties is measurement. Under field conditions other environmental and management effects can mask the distinct influence exerted by plant roots (Bodner et al. 2013). Therefore disturbed soil is often preferred to undisturbed field samples to observe root induced changes under controlled conditions (e.g. Scanlan 2009). In spite of more powerful 3D imaging methods to capture small scale pore processes in recent years (e.g. Gregory et al. 2003; Mooney et al. 2012), still scaling to macroscopic parameters governing the effective hydraulic behavior of soil is challenging. At the macroscopic scale inverse methods have been increasingly used to characterization hydraulic properties from observed state variables in a soil sample (e.g. Hopmans and Šimůnek 1999; Hopmans et al. 2002; Ritter et al. 2003). In spite of being indirect estimates only, these effective parameters can properly describe the hydraulic material properties (water retention, hydraulic conductivity) that underlie an observed the flow behavior. Comparison to direct measurements (pressure plate) showed that they deviated from inverse retention curves mainly towards the dry end (Šimůnek et al. 1998). In the wet range, where soil structural dynamics have key role, there was less deviation. Reliability of inverse estimates also depends on the range of data used for optimization estimation. For example, tension infiltrometers cover a narrow near-saturated pressure head range only (h > −20 to −15 cm; e.g. Schwen et al. 2011). Drainage experiments from large columns typically achieve an intermediate range (h > −150 to −100 cm; e.g. Ritter et al. 2004). Outflow experiments from small sample cylinders can cover a relatively wide pressure head range (h > −800 cm; e.g. Eching and Hopmans 1993). Šimůnek et al. (1998) showed that inverse parameters could better describe flow processes in the natural soil systems compared to lab samples. Furthermore inverse parameters are often obtained from sampling approaches that cover a comparatively high representative elementary volume. This is an important advantage for representative characterization of the highly variable structural range.

From an agricultural point of view biological management of soil structure by roots (“biotilling”) is still at its infancy. Yunusa and Newton (2003) presented the concept of primer-plants, i.e. plants without a direct economic benefit, but effective in conditioning the soil for cash crops and in conserving environmental resources. Currently this type of plants is used as cover crops in agro-environmental programs to minimize nitrate leaching (e.g. Vidal and López 2005) and reduce soil erosion (e.g. Zuazo and Pleguezuelo 2008). Several authors observed cover crop effects on soil structural properties such as aggregate size and stability (Liu et al. 2005) as well as hydraulic processes such as water infiltration (Carof et al. 2007; Bodner et al. 2008). Williams and Weil (2004) showed that cover crops could be an effective way to alleviate soil compaction due to root biopores being used by the following soybean crop to penetrate the soil.

The objective of our study was (i) to identify if there is a short term influence of growing plants on soil hydraulic properties in a soil column experiment, (ii) to analyse which particular pore property and pore range are influenced during the phase of active root growth, and (iii) to model the temporal change of the pore size distribution (PSD) in a non-planted compared to a rooted soil column. Our model plants were two commonly used cover crop species. The focus of the study was on the extent of pore clogging vs. formation of new pores by actively growing roots. We expected higher changes in soil hydraulic properties with more densely rooted soil, and a dominant reduction of pore volume in the pore range corresponding to root diameters. The overall aim was to improve the quantitative understanding of hydrological interactions of roots with soil structural porosity.

## Material and methods

### Column experiment

The influence of plant roots on soil pore properties was measured in a column experiment with planted vs. unplanted soil columns using an inverse evaluation procedure to quantify hydraulic properties.

### Soil column design

**Fig. 1:**
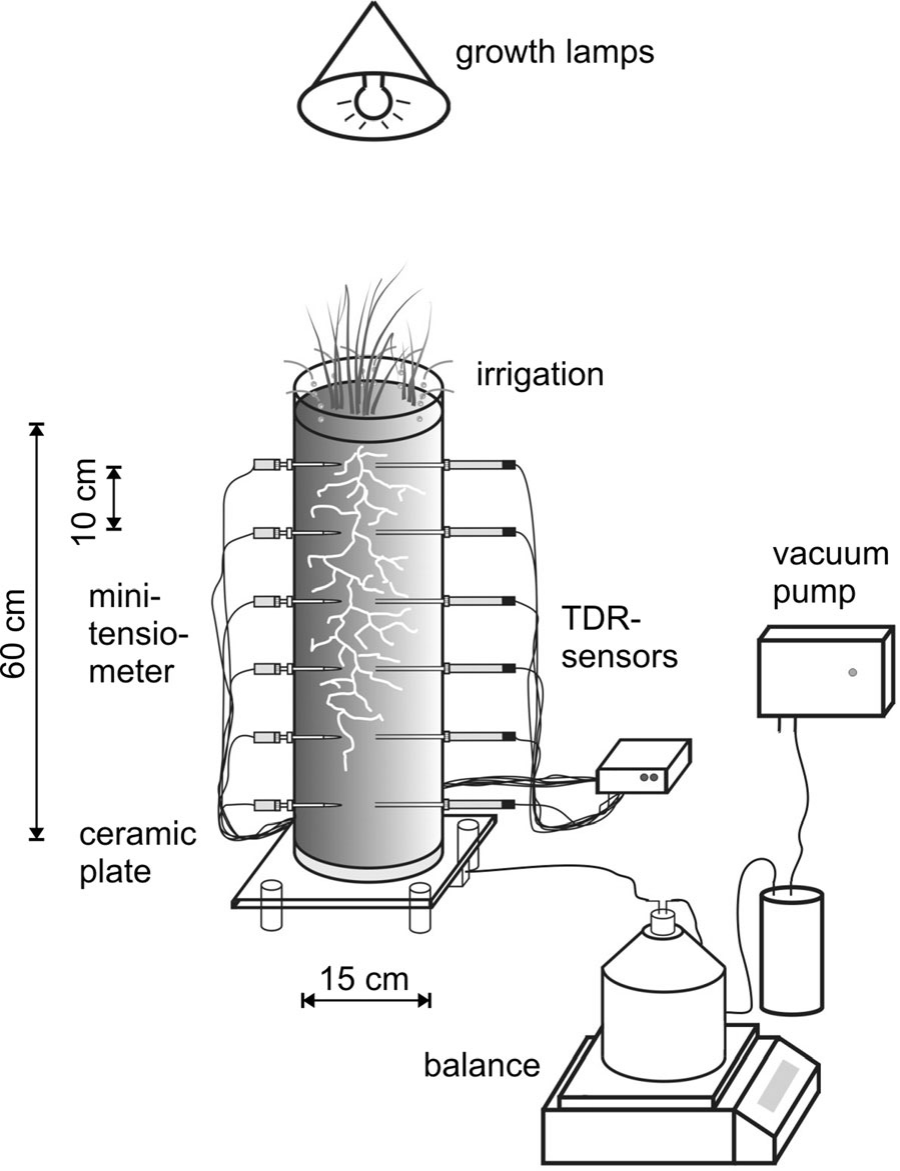
Design of soil columns used for drainage experiments. Each column (Ø 15 cm, height 60 cm) is equipped with six TDR sensors and six tensiometers (LOM, Easy Test) in 10 cm increments, a suction plate (−800 cm air entry) at bottom and micro-drip-irrigation at the top. (Manufacturer of columns, suction plate and drip irrigation: Technisches Büro für Bodenkultur, Austria)

We used twelve cylindrical (60 cm high, 15 cm diameter), custom-built soil columns made of Plexiglas. The column design (Fig. 1) was similar to the one used by Kosugi and Inoue (2002), Ritter et al. (2004) and Yang et al. (2004).

The columns were equipped with TDR probes and mini-tensiometers (LOM, EasyTest, Poland) in six depths separated by 10 cm increments (5, 15, 25, 35, 45 and 55 cm) for continuous measurement of water content and matrix potential. At the top of each column a micro-drip-irrigation was installed to apply a controlled upper boundary flux for drainage experiments and to supply plants with water during the growing period. Irrigation was managed by a PC-controlled multi-channel precision pump with each column supplied by a separate channel. Growth lamps (4 × Narva LT 58 Watt, 8 × Sylvania GroLux F58 Watt) were mounted above the columns to lighten plants. A simple timer controlled lightening duration which was set at 10 h per day.

For controlled drainage, each column was equipped with a porous ceramic suction plate at the bottom to apply a defined lower boundary pressure head condition via different setting of the connected vacuum pump (0 to −800 mbar). From the suction plate a pipe led to a collection bottle (volume: 2,000 cm^3^) placed on a balance for recording the outflow volume in 10 min intervals. Pipes were fixed to a frame in order to avoid unmeant pressure on the balance plates that could bias outflow measurement.

All components of the system (TDR, tensiometers, suction at bottom boundary) were previously calibrated and tested. Products and systems of the various manufacturers (in total 144 sensors) were combined via a self-programmed software based on MS.NET 2.0 for data collection at 10 min intervals. The dataset was finally stored in an MS Access database.

### Experimental conditions

All 12 soil columns were filled with air dried soil sieved to <2 mm. The soil type was a calcareous chernozem with a silt loam texture (0.19 kg kg^−1^ sand, 0.56 kg kg^−1^ silt, 0.24 kg kg^−1^ clay). Organic carbon content of the soil was 0.025 kg kg^−1^. In order to exclude complex interactions between roots and soil micro-organisms, the experiment was performed with sterilized soil. Heat sterilization was done using an autoclave. Preliminary tests did not show differences in early vigour of plants growing in autoclaved and non autoclaved soil. Soil sterilization ensured that root effects were limited to mechanical influences (pore clogging, pore enlargement by root pressure, entanglement of soil particles) and root-induced gluing of soil particles by root exudates.

Column filling was performed carefully to avoid displacement of fine soil particles and layering. A constant bulk density of 1.3 g cm^−3^ was adjusted by successive filling and compaction of 5 cm layers. Boundary compaction was minimized by loosening the contact zone at each filling level. Before sensor installation, columns were fully saturated and drained for three times to limit further soil settlement during the main experimental run. The top of the columns was covered with a two cm layer of fine gravel to avoid surface effects from drip irrigation as well as to reduce evaporation.

After an initial drainage experiment with unplanted columns, mustard (*Sinapis alba* L.) and rye (*Secale cereale* L.) were planted in four replicates, while four columns remained unplanted. These two commonly used cover crops differ in root architecture, mustard having a taproot system and rye a fibrous root system. Plant density followed common seeding rates as used also in a parallel field experiment (Bodner et al. 2013). Three vigorous plants per column were maintained after an initial seeding of six seeds per column. After 3.5 months roots could be observed at the bottom of the columns indicating a sufficiently developed root system. At this time, rye was at BBCH stage 32–33 and mustard at the onset of flowering (BBCH 60–61). After harvest of shoot biomass, a final drainage experiment was performed.

### Measurements

Soil hydraulic properties were determined in a drainage experiment following Kosugi and Inoue (2002) and Ritter et al. (2004). An initial measurement (without root effects) was performed before planting, while the final measurement (with root induced changes) was done after full crop development. The lower boundary during the drainage experiment was set to a constant pressure head of −500 cm. At the upper boundary an irrigation flux of 5 mm h^−1^ was applied until a steady outflow rate was achieved as initial condition. Thereafter irrigation was stopped and water content, pressure head and outflow rate were monitored during the redistribution phase. Soil surface was covered by parafilm to avoid evaporation (i.e. no flux upper boundary condition), while at the lower boundary drainage continued under a constant pressure head of −500 cm. The transient profiles of water content, pressure head and outflow during the redistribution phase entered the inverse evaluation procedure for hydraulic property estimation. Data recording was stopped after about 100–110 h when outflow rates had approached zero and changes in water content over a period of 6 h were as low as 0.001 cm^3^ cm^−3^. A second drainage experiment was performed subsequently to obtain a validation data set. Columns were again irrigated to the initial steady outflow rate and drained under the same conditions as described previously.

After the end of the experiment, soil was removed from the columns and root morphological parameters were measured at 10 cm increments. Root analysis followed the procedure described by Himmelbauer et al. (2004). Roots were washed free from soil over a set of sieves up to 0.5 mm with tap water, stained and thereafter analyzed using WinRhizo (Regent Instruments Inc.). Finally root and aboveground dry matter were measured after oven-drying at 60 °C until constant weight.

### Determination of hydraulic properties

Determination of soil hydraulic properties was done by inverse parameter estimation using HYDURS 1D v. 4.16 (Šimůnek et al. 2013). The procedure is described in detail by Hopmans et al. (2002). In short, parameters for the constitutive relations - soil water retention S_e_(h) and hydraulic conductivity K(h) – in a 1D vertical water flow simulation via Richards’ equations are obtained by minimizing the deviation between observed and simulated state variables. We used the model of Kosugi (1996) to describe S_e_(h) and K(h) which is based on a lognormal pore-size distribution. S_e_(h) is given by

1 $$ {S}_e(h)=0.5 erfc\left(\frac{ \log \left(\frac{h}{h_m}\right)}{\sqrt{2\sigma }}\right) $$

where S_e_ (−) is the effective saturation corresponding to $\frac{\theta -{\theta}_r}{\theta_s-{\theta}_r}$ with *θ*_*r*_ (cm^3^ cm^−3^) being residual water content and *θ*_*s*_ (cm^3^ cm^−3^) saturation water content. *Erfc* is the complementary error function, *h*_*m*_ (cm) the median pressure head and *σ* (−) the standard deviation of the log-transformed pressure head. K(h) can be written as

2 $$ K(h)=\left\{\begin{array}{cc}\hfill {K}_s{S_e}^l{\left\{\frac{1}{2} erfc\left[\frac{ \ln \left(\frac{h}{h_m}\right)}{\sqrt{2\sigma }}+\frac{\sigma }{\sqrt{2}}\right]\right\}}^2\hfill & \hfill \left(h<0\right)\hfill \\ {}\hfill {K}_s\hfill & \hfill \left(h\ge 0\right)\hfill \end{array}\right. $$

where *K*_*s*_ (cm s^−1^) is saturated hydraulic conductivity and *l* (−) is a tortuosity factor.

Observed state variables for inverse optimization were the transient time series of water content, pressure head and cumulative outflow during the redistribution phase in six soil layers and 10 min interval. Simulated values of these variables were obtained by numerically solving Richards’ equation in HYDURS 1D with a no flux upper boundary and a constant head (−500 cm) lower boundary condition. The total of differences between measured and simulated values are expressed by the objective function being

3 $$ \varPhi \left(\beta, y\right)={\displaystyle \sum_{j=1}^{j={m}_y}{v}_j}{{\displaystyle \sum_{i=1}^{i={n}_j}{w_i}_j\left[{y}_j\ast \left(z,{t}_i\right)-{y}_j\left(z,{t}_i,\beta \right)\right]}}^2 $$

where the right side represents residuals between measured (*y*_j_*) and corresponding predicted (*y*_*j*_) variables using the soil hydraulic parameters of the optimized parameter vector *β*. Measured state variables are denoted by *m*_*y*_, whereas the number of measurements for a certain state variable are given by the variable *n*_*j*_. Weighing factors *v*_*j*_ and *w*_*ij*_ can be included for a given state variable or an individual data point respectively. For our study, the total number of data points in the objective function was between 7,600 and 7,900. The inverse problem was solved by minimizing the objective function using the nonlinear Levenberg-Marquardt optimization algorithm implemented into HYDRUS 1D.

HYDRUS 1D allows a maximum number of 15 parameters to be optimized simultaneously. Still it is recommended to reduce the number of parameters to avoid non-unique solutions (e.g. Durner et al. 1999; Abbasi et al. 2003a, b). Therefore we subdivided the soil column into two layers only (0–30 cm, 30–60 cm), assuming distinct temporal changes between the more densely rooted upper part and the less rooted lower part of the columns. Residual water content was set to 0.067 and the tortuosity parameter *l* was set to 0.5 for all simulations as predicted by the texture based pedotransfer function Rosetta (Schaap et al. 2001). In this way a total number of eight parameters (*θ*_*s*_, *h*_*m*_, *σ* and *K*_*s*_ for two layers) had to be optimized. Initial parameter estimates were obtained from direct fitting of the Kosugi retention model to data-pairs of water content and pressure head using RETC (van Genuchten et al. 1991). Initial estimates of *K*_*s*_ were also taken from Rosetta.

In order to ensure a unique solution (global minimum) we changed the initial values and assessed whether the final estimates converged at the same values. Furthermore we checked the statistical parameters (standard error coefficient, confidence limits, R^2^ between measured vs. predicted) provided by HYDRUS 1D to ensure reliability of estimation results. Finally also statistical analysis of the obtained parameter values provides an indicator for the quality of optimization results. In case of a non-unique solution, error variance would increase by an additional random effect due to different optimization quality, while the relative weight of the fixed factor effects (e.g. plant influence) would decrease. Thus also statistical significance indirectly reveals the reliability of the parameter optimization results.

### Simulation of pore evolution

The pore evolution model presented by Or et al. (2000) was tested to describe the root induced changes of hydraulic properties. Pore size distribution (PSD) *f* is the first derivative of the retention curve and can be written as

4 $$ f(r)=\frac{\theta_s-{\theta}_r}{\sigma r\sqrt{2\pi }} \exp \left\{-\frac{{\left[ \ln \left(r/{r}_m\right)\right]}^{{}^2}}{2{\sigma}^2}\right\} $$

where *r* (μm) is the pore radius, *r*_*m*_ (μm) is the median pore radius, and *σ* (−) is its standard deviation. The median pore radius (*r*_*m*_) can be calculated from the median pressure head *h*_*m*_ using the Young-Laplace equation.

According to the pore evolution model of Or et al. (2000) the temporal change of the PSD is given by

5 $$ \frac{\partial f}{\partial t}=\frac{\partial }{\partial r}\left(D\left(r,t\right)\frac{\partial f}{\partial r}\right)-\frac{\partial }{\partial r}\left(V\left(r,t\right)f\right)-M(t)f $$

where *t* is time, *V* (μm s^−1^) is a drift term, *D* (μm^2^ s^−1^) a dispersion term and *M* (s^−1^) a degradation term. The drift and dispersion terms quantify changes with time of *r*_*m*_ and σ respectively. *M* represents a sink term for changes in total porosity. Dispersion is related to drift by a constant dispersivity *λ* (μm). An analytical solution of Eq. 5 was derived by Leij et al. (2002). To model the distinct temporal changes of the PSD between the initial and final state of the three treatments (no plant, mustard, rye) we optimized the cumulative drift term *T* (i.e. the integral of *V*; *cf*. Leij et al. 2002) as well as dispersivity. *M* was set equal the reduction in total porosity. While other authors limited degradation to the macropore range (e.g. Schwärzel et al. 2011), due to the lack of proper data, we did not attribute degradation to any distinct pore range.

After testing the model for the temporal changes between the initial and final state, we simulated the case of pore evolution with increasing rooting density based on the data from the final experiment. For this purpose we used a logistic function following Leij et al. (2002) to describe the drift term as a function of root length density while dispersivity was again assumed as constant over time. All calculations of pore evolution were done with Matlab Version 8 R2012b.

### Statistical evaluation

Statistical data evaluation (hydraulic parameters, plant data) was performed by analysis of variance with the procedure PROC MIXED in the software SAS 9.2. This procedure is based on restricted maximum likelihood estimates of the variance components (Littell et al. 1998) and provides Wald-type F-statistics using GLSE (generalized least squares). A mixed model for performing a proper analysis of variance is required as our data include repeated measures over depth (two layers) and time (initial and final experimental run). Thus an adequate correlation model has to be fit to account for serial correlation of non-randomized repeated measurements on the same experimental unit (Piepho et al. 2004). According to the Akaike Information Criterion (AIC) an unstructured (UN) model fitted best to our data. In case of significant effects at *p* < 0.05 in the analysis of variance, comparison of means was performed using a two-sided t-test.

## Results

### Plant development

Shoot growth of the plants grown in the columns achieved dry matter values of 9.19 ± 1.03 g for mustard and 1.40 ± 0.22 g for rye. This would correspond to 5,200 kg ha^−1^ dry matter for mustard and 792 kg ha^−1^ for rye, being in the range of field measured values (e.g. Bodner et al. 2010, 2013). While mustard plants had a vigorous aboveground growth, tillering of rye was less compared to field grown plants, resulting in a lower biomass than expected at the end of the experiment (BBCH 32–33).

**Fig. 2:**
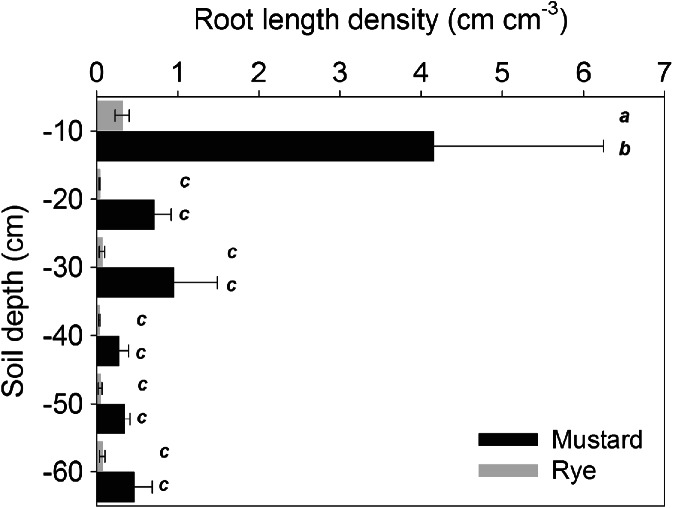
Root length density distribution in the soil columns of mustard and rye in six layers (0 to 60 cm) determined by image analysis. (*Bars* with the same letter indicate non-significant differences at *p* < 0.05)

Figure 2 shows root length density of the two species at the end of the experiment. Root length density of mustard was significantly higher compared to rye in the upper soil layer. Visual inspection during root analysis showed that the extension of the mustard tap root was around 10 cm, i.e. within the uppermost soil layer. Generally both plants had a lower rooting density compared to plants grown under field conditions (e.g. Bodner et al. 2010).

Rye had a significantly higher root diameter than mustard in all depth. Root diameters of both species (mustard 0.27 mm, rye 0.34 mm) were in the range of values measured under field conditions (Bodner 2007). Within each species there was no significant decrease of root diameter over column depth.

Total root volume was 6.36 ± 1.40 cm^3^ for mustard and 0.79 ± 0.02 cm^3^ for rye. Scanlan and Hinz (2010) used a lognormal function to study species differences in root volume distribution over different diameter classes. Our sample showed a median root diameter of the lognormal distribution at 0.21 mm for mustard and 0.16 mm for rye, and a standard deviation of the distribution of 0.94 for mustard and 0.72 for rye respectively. The higher median diameter of mustard shows that in spite of the slightly smaller average diameter, coarse root segments, particularly the tap root, substantially contributed to total root volume. The higher standard deviation of mustard indicates a more even contribution of coarse and root fine axes to total root volume. The narrower distribution for rye reveals that here root volume was built by morphologically less differentiated axes types.

### Estimation of soil hydraulic properties

Table 1 shows the direct parameter estimates obtained by fitting the Kosugi retention model to data pairs of water content and pressure head using RETC.

**Table 1:** Initial estimates (means ± standard deviation) of the parameters of Kosugi’s retention model obtained by curve fitting to data pairs of water content and pressure head using RETC

Treatment	Depth cm	θ_s_ cm^3^ cm^−3^	h_m_ cm	σ −	R^2^
Initial drainage experiment (unplanted columns)					
Unplanted ↳ No plant*	0–30	0.38 (<0.01)	284.7 (±18.2)	2.15 (±0.11)	0.99 (<0.01)
	30–60	0.39 (<0.01)	388.8 (±35.3)	1.99 (±0.11)	0.97 (<0.01)
Unplanted ↳ Mustard	0–30	0.39 (<0.01)	272.8 (±22.5)	2.22 (±0.14)	0.97 (±0.01)
	30–60	0.40 (<0.01)	302.0 (±24.9)	1.89 (±0.10)	0.95 (±0.01)
Unplanted ↳ Rye	0–30	0.37 (±0.01)	308.2 (±25.3)	2.22 (±0.07)	0.98 (<0.01)
	30–60	0.39 (±0.01)	352.3 (±37.3)	2.06 (±0.08)	0.96 (±0.01)
Final drainage experiment (unplanted vs. planted columns)					
No plant	0–30	0.38 (<0.01)	466.3 (±48.1)	2.44 (±0.16)	0.96 (±0.01)
	30–60	0.38 (<0.01)	491.5 (±35.4)	2.26 (±0.17)	0.98 (<0.01)
Mustard	0–30	0.39 (<0.01)	356.6 (±60.6)	2.49 (±0.16)	0.94 (±0.04)
	30–60	0.38 (<0.01)	265.2 (±20.6)	1.87 (±0.10)	0.98 (<0.01)
Rye	0–30	0.39 (±0.01)	540.0 (±63.3)	2.82 (±0.12)	0.94 (±0.01)
	30–60	0.38 (±0.01)	488.6 (±82.4)	2.51 (±0.30)	0.99 (<0.01)

*For the pre-planting initial drainage experiment ↳ indicates the subsequent treatment

**Fig. 3:**
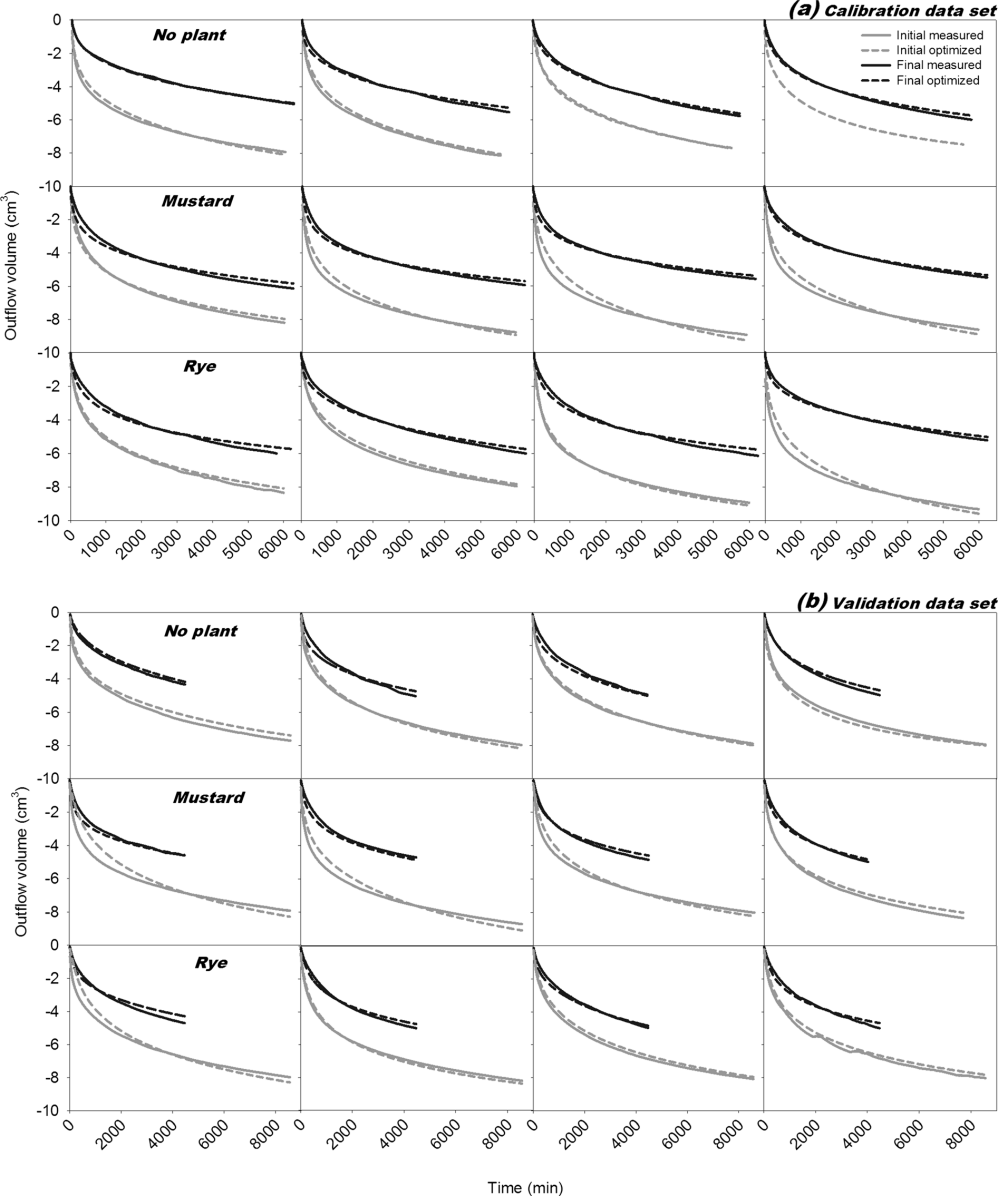
Measured and simulated cumulative outflow for **a** The calibration data set and **b** The validation data set. The calibration data were used to inversely estimate soil hydraulic property parameters, while the simulated outflow for the validation data was obtained in a forward simulation using the same hydraulic parameters. All simulations were done with HYDRUS 1D

These direct estimates were then used as starting values for inverse optimization of the Kosugi parameters in each soil column. Table 2 gives the obtained parameter values. Additionally Fig. 3a and b show measured and simulated outflow for the calibration and validation data sets.

**Table 2:** Parameters (means ± standard deviation) of Kosugi’s hydraulic property model obtained by inverse estimation using HYDURS 1D

Treatment	Depth cm	θ_s_ cm^3^ cm^−3^	h_m_ cm	σ −	K_s_ cm min^−1^	R^2^	SSQ
Initial drainage experiment (unplanted columns)							
Unplanted ↳ No plant*	0–30	0.46 (±0.02)	90.1 (±19.7)	2.17 (±0.10)	1.22 (±0.44)	0.93	0.39
	30–60	0.46 (±0.01)	296.9 (±42.3)	2.47 (±0.04)	0.97 (±0.34)		
Unplanted ↳ Mustard	0–30	0.42 (±0.01)	142.4 (±26.3)	1.78 (±0.06)	0.28 (±0.10)	0.94	0.82
	30–60	0.48 (±0.01)	142.2 (±6.9)	2.33 (±0.15)	23.97 (±11.91)		
Unplanted ↳ Rye	0–30	0.40 (±0.01)	159.1 (±15.9)	1.79 (±0.03)	0.19 (±0.04)	0.90	0.67
	30–60	0.46 (±0.02)	145.3 (±28.4)	2.08 (±0.05)	0.37 (±0.14)		
Final drainage experiment (unplanted vs. planted columns)							
No plant	0–30	0.39 (±0.002)	162.2 (±13.1)	1.33 (±0.08)	0.11 (±0.03)	0.98	0.34
	30–60	0.41 (±0.01)	334.5 (±21.3)	2.71 (±0.11)	3.11 (±1.44)		
Mustard	0–30	0.43 (±0.01)	207.3 (±18.2)	2.85 (±0.03)	21.46 (±5.27)	0.97	0.46
	30–60	0.40 (±0.002)	432.4 (±31.5)	3.42 (±0.07)	28.68 (±7.92)		
Rye	0–30	0.41 (±0.002)	230.4 (±28.6)	2.43 (±0.23)	29.35 (±9.29)	0.98	0.42
	30–60	0.40 (±0.01)	474.1 (±34.4)	3.13 (±0.14)	26.24 (±9.12)		

*For the pre-planting initial drainage experiment ↳ indicates the subsequent treatment

With only some exceptions a high overall R^2^ (Table 2) between observed and predicted data was obtained. This is shown for cumulative outflow in Fig. 3a, b. Data of simulated and measured water content and pressure head time series in the six observation depths are not shown. The overall optimization R^2^ did not show significant differences between the initial and final experiment in spite of an average higher R^2^ for the final run. Importantly there was no influence of plant treatments on the optimization R^2^. Also the standard errors of optimized parameters were low. On average the relative standard error (i.e. mean/standard error) for θ_s_ was 0.53 %, for h_m_ 3.0 % and for σ 1.6 %. Only K_s_ had a higher standard error (22.1 %) indicating highest uncertainty for this parameter.

For outflow profiles further statistical indicators for the quality of estimated vs. measured data were determined (Table 3) using the IRENE software (Fila et al. 2003). They also suggested a generally good fit between measured and simulated data and thus reliable hydraulic parameter estimates obtained by optimization. A slightly lower quality of fit was obtained for the validation compared to the calibration data, although this difference was statistically significant only for the relative mean error (4.0 % vs. 6.1 %). Again no indicator showed any significant difference between plant treatments. Thus fitting criteria demonstrated that optimization induced no systematic bias for treatment comparison.

**Table 3:** Statistical indicators for the quality of the estimated hydraulic properties

		Calibration		Validation	
		Initial	Final	Initial	Final
No plant	Mean error (%)	2.43 (±0.60)	3.41 (±1.92)	3.50 (±1.57)	8.03 (±3.03)
	RMSE (cm^3^)	0.15 (±0.05)	0.12 (±0.06)	0.20 (±0.09)	0.21 (±0.05)
	CD (−)	1.02 (±0.24)	0.84 (±0.10)	1.08 (±0.14)	0.83 (±0.17)
Mustard	Mean error (%)	4.27 (±0.85)	4.79 (±1.52)	5.57 (±3.77)	6.92 (±2.05)
	RMSE (cm^3^)	0.29 (±0.09)	0.17 (±0.06)	0.39 (±0.29)	0.19 (±0.03)
	CD (−)	1.29 (±0.34)	0.76 (±0.07)	1.50 (±0.36)	0.81 (±0.08)
Rye	Mean error (%)	4.21 (±1.79)	5.43 (±0.57)	4.79 (±3.39)	7.44 (±2.29)
	RMSE (cm^3^)	0.18 (±0.01)	0.19 (±0.04)	0.31 (±0.22)	0.22 (±0.07)
	CD (−)	1.01 (±0.03)	0.75 (±0.03)	1.41 (±0.34)	0.71 (±0.08)

Calculations are based on the agreement between measured and modelled cumulative outflow for the calibration and validation data sets (RMSE is root mean square error and CD is coefficient of determination with 1 showing optimum fit)

The reduction in average total porosity between initial and final experiments (mean of all treatments at initial experiment: 0.45 cm^3^ cm^−3^, mean of all treatments at final experiment: 0.41 cm^3^ cm^−3^; *cf*. Table 2) indicates soil settlement over time. Also mean *h*_*m*_ decreased in the course of the experiment from −162.7 to −306.8 cm indicating a shift to a smaller pore size, while σ increased from an average of 2.10 at the beginning to 2.65 at the end. Interestingly there was a significant increase in *K*_*s*_ between the initial and the final experiments in spite of a reduced *θ*_*s*_ and *h*_*m*_. Among Kosugi pore parameters, pore radius standard deviation showed the strongest relation to *K*_*s*_ (r^2^ = 0.57 and 0.50 for the initial and final experiment, respectively). Thus in spite of the decrease in *h*_*m*_ and the related shift of median pore radius to smaller pore classes, the broader range of different pore radius classes indicates the formation of additional macroporosity. These pores of large diameter can essentially contributed to *K*_*s*_ due to high transport capacity (Poisseuille’s law) and lower tortuosity (Vervoort and Cattle 2003).

### Plant root influence on pore size distribution

Table 4 shows the results of analysis of variance highlighting which factors significantly influenced the inversely determined hydraulic parameters. The corresponding parameter values are given in Table 2.

**Table 4:** Significance (*p*-values) of factors underlying the differences in soil hydraulic parameters calculated by a mixed model analysis of variance (* *p* < 0.05, ** *p* < 0.01, *** *p* < 0.001, *ns* not significant)

	θ_s_ cm^3^ cm^−3^	h_m_ cm	σ −	K_s_ cm min^−1^
Time (T)	0.003**	0.005**	0.003**	<0.001***
Depth (D)	0.255 *ns*	0.003**	<0.001***	0.554 *ns*
Plant (P)	0.416 *ns*	0.702 *ns*	0.078 *ns*	0.074 *ns*
T*D	0.013*	0.683 *ns*	0.040*	0.829 *ns*
T*P	0.345 *ns*	0.886 *ns*	0.006**	<0.001***
D*P	0.985 *ns*	0.139 *ns*	0.514 *ns*	0.783 *ns*
T*D*P	0.036*	0.167 *ns*	0.165 *ns*	0.432 *ns*

A significant influence of plant treatments was found for σ and K_s_. Plant treatments had a significant interaction with time, i.e. differences were only relevant for the final experimental run (after root growth) as expected. For both parameters, treatments with plants (*σ* = 2.96; *K*_*s*_ = 26.4 cm min^−1^) differed from the non-planted control (*σ* = 2.02; *K*_*s*_ = 1.6 cm min^−1^), while planted columns were similar among each other. The temporal change of these parameters between the initial and final experiment was not significant for the unplanted columns (*σ*_*initial*_ = 2.32 vs. σ_final_ = 2.02; *K*_*s*,*initial*_ = 1.1 cm min^−1^vs. *K*_*s*,*final*_ = 1.6 cm min^−1^), while they changed significantly for the planted ones (*σ*_*initial*_ = 2.00 vs. *σ*_*final*_ = 2.96; *K*_*s*,*initial*_ = 6.2 cm min^−1^vs. *K*_*s*,*final*_ = 26.4 cm min^−1^).

For saturation water content plant treatments showed significant interaction with time and depth. Comparison of means revealed that a plant effect could be demonstrated only for the final experiment and the more densely rooted upper layer. Again the planted treatments (mustard 0.43 cm^3^ cm^−3^, rye 0.41 cm^3^ cm^−3^) differed significantly from the non-planted soil (0.39 cm^3^ cm^−3^) while being similar among each other. For *θ*_*s*_ temporal dynamics showed a significant reduction between the initial (0.47 cm^3^ cm^−3^) and final experiment (0.40 cm^3^ cm^−3^) in the lower layer for all treatments, while in the upper layer only the bare soil treatment showed a significant pore loss. The planted columns obviously reduced the decrease in *θ*_*s*_ via roots stabilizing the pore system against further settlement.

*H*_*m*_ was not significantly changed by plant roots and only differed in average between the initial (−162.7 cm) and final experiment (−306.8 cm) as well as between the two layers (*h*_*m*,*upper*_ = 165.3 cm vs. *h*_*m*,*lower*_ = 304.2 cm).

**Fig. 4:**
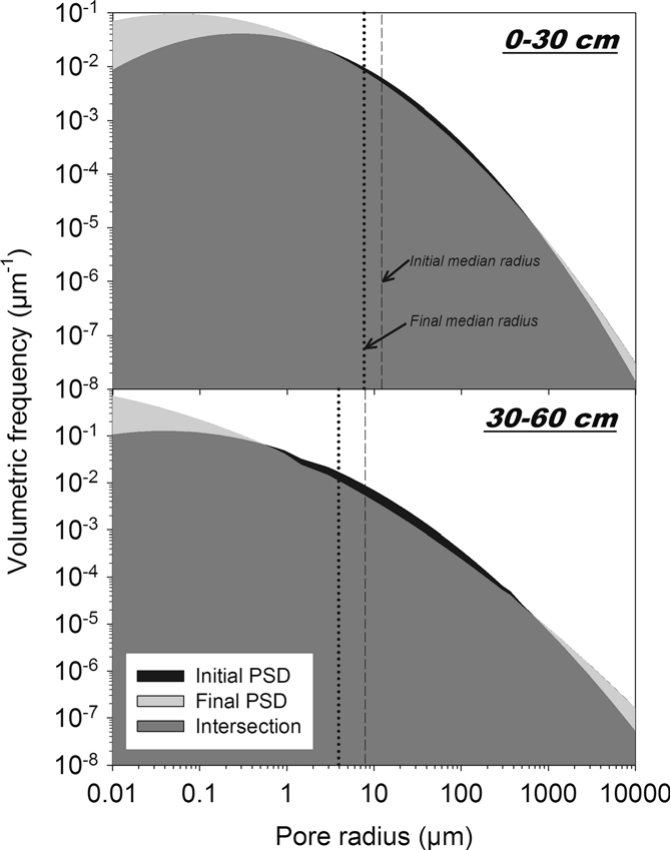
Pore size distribution (log-log scale) in the upper (0–30 cm) and lower (30–60 cm) layers of the soil columns at the initial (before planting) and final (with plant influence) state. Temporal changes in volumetric frequency at different pore radius classes are highlighted (*black colour*: decreasing frequency, *light grey colour*: increasing frequency)

**Fig. 5:**
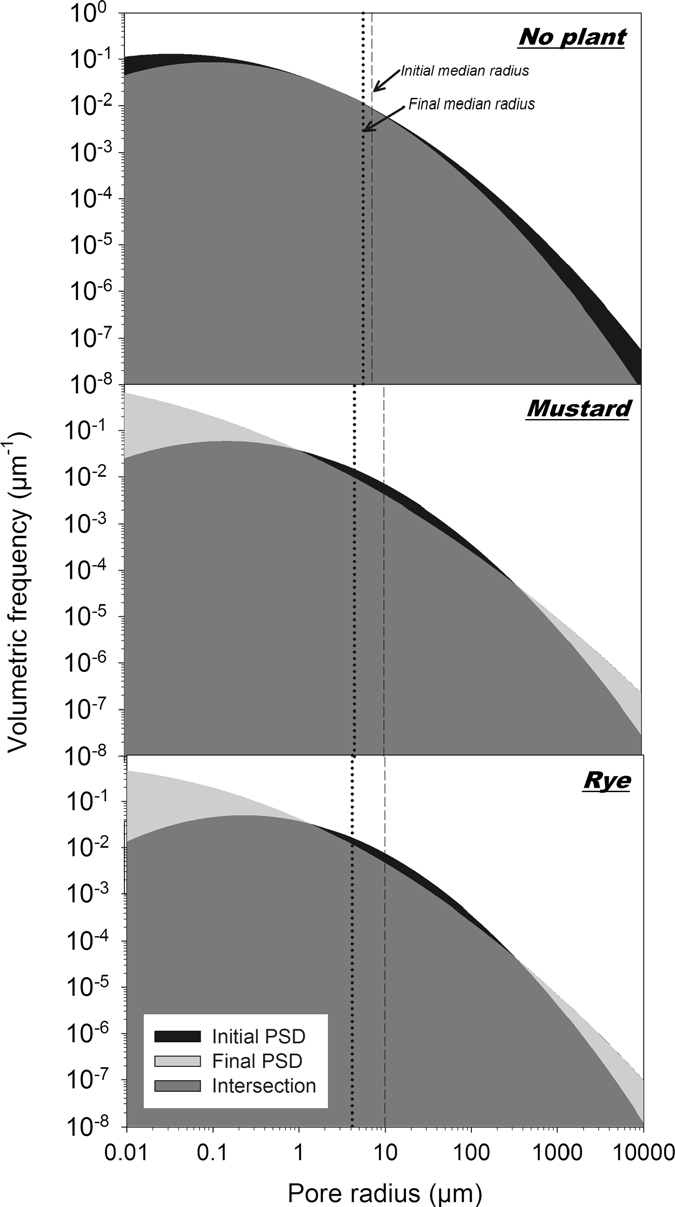
Pore size distribution (log-log scale) of the non-planted and planted (mustard, rye) soil columns at the initial (before planting) and final (with plant influence) state. Temporal changes in volumetric frequency at different pore radius classes are highlighted (*black colour*: decreasing frequency, *light grey colour*: increasing frequency)

Changes in overall PSD between the initial and final experiment for the two depths (i.e. interaction of T*D) and the different treatments (i.e. interaction of T*P) are shown in Figs. 4 and 5. Tables 5 and 6 give the respective volumetric changes for different pore classes using the classification of the Soil Science Society of America (SSSA 2013). This classification defines macropores having a radius >37.5 μm (37.5≤ *r* < 500 μm very fine macropores), mesopores between 37.5 μm and 15 μm, and micropores <15 μm (*r* < 2.5 μm ultramicropores and cryptopores).

**Table 5:** Temporal change of pore volume (mean of planted and unplanted columns) at different pore classes according to SSSA (2013) between initial and final drainage experiment in the upper (0–30 cm) and lower (30–60 cm) layers of the soil columns

	Volume change *cm* ^*3*^ *cm* ^−*3*^	
	Upper layer	Lower layer
Micropores1 (*r* < 2.5 μm)	0.0304	0.0274
Micropores2 (2.5≤ *r* < 15 μm)	−0.0146	−0.0451
Mesopores (15≤ *r* < 37.5 μm)	−0.0136	−0.0230
Macropores1 (37.5≤ *r* < 500 μm)	−0.0167	−0.0269
Macropores2 (r ≥ 500 μm)	0.0011	0.0049

**Table 6:** Temporal change of pore volume at different pore classes according to SSSA (2013) between initial and final drainage experiment for the non-planted and planted (mustard, rye) soil columns

	Volume change *cm* ^*3*^ *cm* ^−*3*^		
	No plant	Mustard	Rye
Micropores1 (*r* < 2.5 μm)	−0.0120	0.0543	0.0567
Micropores2 (2.5≤ *r* < 15 μm)	−0.0031	−0.0463	−0.0410
Mesopores (15≤ *r* < 37.5 μm)	−0.0070	−0.0267	−0.0246
Macropores1 (37.5≤ *r* < 500 μm)	−0.0268	−0.0259	−0.0219
Macropores2 (*r* ≥ 500 μm)	−0.0093	0.0123	0.0070

There was a general decrease in pore volume over time in both layers (upper: −0.013 cm^3^ cm^−3^; lower −0.063 cm^3^ cm^−3^). As shown in Table 5, this was the result of a reduction in a pore range between 2.5 and 500 μm (micropores to very fine macropores). An increase was registered for micropores <2.5 μm and macropores ≥500 μm. The lower layer showed 79 % higher overall loss of pores while increase in pores <2.5 μm and ≥500 μm was similar to the upper layer. Total pore loss in the lower layer was 13.5 % of initial porosity, while in the upper layer it was only 3.1 %.

There was a clear differentiation in pore dynamics between the planted and unplanted columns (Fig. 5, Table 6).

The reduction in soil porosity for the non-planted columns covered the whole range of pores. Total loss of porosity was −0.058 cm^3^ cm^−3^ increasing towards the range of macropores and finer micropores <2.5 μm. The rooted columns on the contrary, showed a reduction in the range of larger micropores to very fine macropores (2.5 to 500 μm), while increasing in the finer micopore classes <2.5 μm as well as in larger macropores ≥500 μm. Reduction of larger micropores and mesopores was substantially higher in the planted in relation to the unplanted columns, while reduction in very finer macropores was similar. Mustard reduced total porosity to a higher extent (−0.032 cm^3^ cm^−3^) compared to rye (−0.024 cm^3^ cm^−3^). The main differentiation between the planted treatments was in the volumetric increase in larger macropores >500 μm. Here mustard had a 75 % higher increase than rye.

### Simulation of root induced pore evolution

**Fig. 6:**
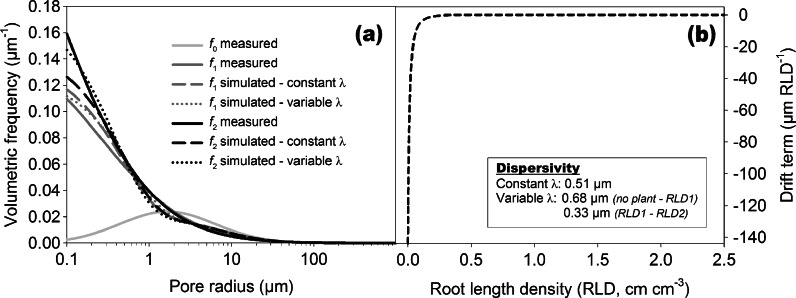
Measured (*solid lines*) and simulated (*short dashed lines*) pore size distributions (PSD) of non-planted and planted soil columns (Mean and 95 % confidence bands). Simulation was done by a pore evolution model calculating the temporal change between an initial (*grey line*) and final state (*black lines*)

Optimization of the governing parameters in the pore evolution model of Or et al. (2000) resulted in a cumulative drift of 15.35 μm and a dispersivity of 1.91 μm for the non-planted treatment. For the planted treatments the obtained parameters were 4.20 and 0.38 μm for mustard and 2.45 and 0.42 μm for rye, respectively. Measured and predicted evolution of PSD is shown in Fig. 6.

Although the simulated PSDs suggest a satisfactory prediction by the model, for some cases the approach was not appropriate. Particularly the non-planted treatment showed a reduction in σ over time in most cases, i.e. a tendency to a narrower PSD. This could not be reproduced by a model based on a dispersion like process of pore evolution, resulting in a high residual error in optimization and therefore unreliable parameter estimates.

**Fig. 7:**
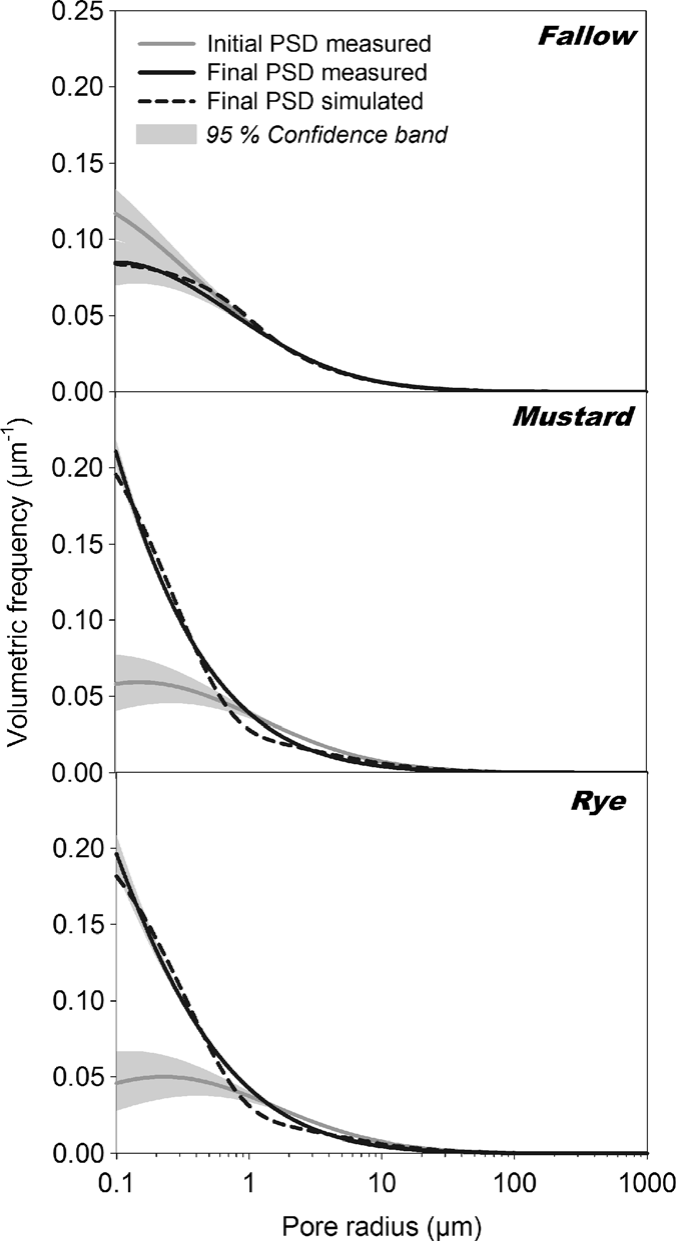
**a** Measured (*solid lines*) and simulated (*long dashed and dotted lines*) pore size distributions (PSD) showing the transition of a non-rooted to an increasingly rooted soil. Simulation was done with a pore evolution model using constant (*long dashed lines*) and variable (*dotted lines*) dispersivity. **b** Governing relations (drift function and dispersivity) for the pore evolution model

Our measurements showed pore evolution towards a broader PSD in planted columns being expressed by an increase in *σ*. This differentiation was significant between non-rooted (*σ* = 2.02) and rooted soil. The trend to higher *σ* in more densely rooted soil of mustard (*σ* = 3.13) compared to rye (*σ* = 2.78) was not significant. This dispersion like dynamics allowed application of the pore evolution model. Figure 7a shows measured and modelled changes in PSD between a non-rooted (RLD_0_ = 0 cm cm^−3^) and a rooted soil with increasing root length density. For this example we assumed that the distinct root influence of the two species was only due to their different rooting density (RLD_1_ = 0.14 cm cm^−3^, RLD_2_ = 1.93 cm cm^−3^).

For this simulation we did not optimize the cumulative drift *T*, but we used a drift term *V* as a logistic function of root length density (Fig. 7b) following Leij et al. (2002). The sharp increase of the drift function to a maximum reveals that there was an immediate reduction in median pore radius between the non-rooted (*r*_*m*_ = 7.2 mm) to the rooted (*r*_*m*_ = 5.7 mm) soil even at low root length density. The subsequent drift induced by higher root length densities was insignificant. The dispersion term *D*, driving changes in σ, is linked to *V* via *D* = λ_*_ *V*. The analytical solution of Eq. 5 developed by Leij et al. (2002) requires a constant dispersivity *λ*. Figure 7a demonstrates that there is an increasing deviation of the modelled PSD with higher root length density. This is the result of assuming a constant *λ* and thereby a functionally similar change of σ with RLD as used for the drift *V*. However measurements demonstrated that there was a trend towards higher s with increasing RLD between the two species. This required a distinctive *λ* for each predictive step which resulted in an improved model fit. This indicates that the linkage between *V* and *D* is problematic in case of root driven pore evolution because of qualitatively and/or quantitatively distinctive changes in *r*_*m*_ and *σ*.

## Discussion

Roots are a key factor in soil aggregation and formation of structural porosity (Angers and Caron 1998). The present study analyzed root influences on PSD during active root growth. The main objective was the quantitative assessment of changes in PSD induced by a tap rooted dicot (mustard) and a fibrously rooted monocot species (rye) compared to a non-planted control.

### Plant development

Root development in the columns achieved lower density compared to field grown plants. This might be related to the unstressed conditions in the columns with optimum water supply and regular addition of nutrient solution during the experiment. De Willigen et al. (2000) reported that a rooting density between 0.5 and 1.0 cm cm^−3^ might be sufficient for optimum plant supply under non-limiting conditions. The moderate root development of rye, with root length density below 1.0 cm cm^−3^ in all layers, however is also related to low tillering and thus little development of shoot-born roots.

Beside maximum rooting density, the two species also differed in average root diameter. Root effects on soil porosity are strongly related to root diameter. While coarser roots have higher strength to shift soil particles due to lower tendency of root buckling under mechanical stress (Bengough et al. 2011), finer lateral roots can access smaller sized pores. Using a lognormal distribution model of root volume over different root diameter classes (Scanlan and Hinz 2010) we highlighted the different volume allocation between the tap rooted and fibrous rooted species: a higher standard deviation of the distribution for mustard pointed to an even allocation of root volume to coarse (tap root, first order laterals) and fine axes (higher order laterals). The median root radius of mustard was 56 % higher than its average radius, while for rye there was hardly a difference between both values. This reveals the important contribution of the tap root to overall root volume for mustard. Morphological differentiation between root axes of rye was less (narrow standard deviation), i.e. the bulk of root volume was formed by axes of similar diameter. Beside the generally more homogeneous diameter distribution in monocot root systems, this distribution pattern also expressed the dominance of primary and basal axes with similar morphology (Kutschera et al. 2009). Coarser shoot-born axes were hardly developed because of reduced tillering.

From root characterization we could expect stronger overall impact on soil porosity by mustard due to higher rooting density. The lower average diameter and larger standard deviation of root volume distribution of mustard (allocation to fine axes) pointed to higher potential of root in-growth and pore clogging. Macropore effects could be expected from coarser rye axes (higher average diameter) as well as the tap root of mustard.

### Soil hydraulic property determination

Parameters of the soil PSD were obtained by inverse estimation from a drainage experiment. Interestingly only for the initial experiment (unplanted columns) there was a significant relation between the directly determined starting values (Table 1) and the final inverse estimates (Table 2), while for the final experiment (planted columns) parameter values did not correlated. Direct parameter estimation from a drainage experiment with large soil columns involves substantial uncertainty due to extrapolation from a rather narrow, near saturated range of measured data towards the entire retention curve (Ritter et al. 2004). In our case the pressure head range was between 0 cm and a minimum at the lowest tensiometer (55 cm depth) of −235.4 cm for the initial experiment and −149.9 cm for the final experiment. The narrower range of θ-h data pairs in the final experiment might have decreased the reliability of direct estimation. Also enhanced soil structuring over time might have been captured appropriately only by the inverse procedure, explaining the lack of correlation between the two procedures at the final experiment.

Most work on inverse estimation of soil hydraulic properties has been performed on small sample cylinders (e.g. Durner et al. 1999; Durner and Iden 2011). Experiments in small samples (e.g. one-step, multi-step outflow) cover a wider range of θ and h compared to larger volumes (e.g. soil columns, lysimeters). Also equilibration of the system to an applied boundary pressure head is achieved more readily. The pressure head range reported from studies using soil columns for inverse parameter estimation was from saturation to about −100 cm (e.g. Kosugi and Inoue 2002; Ritter et al. 2004; Yang et al. 2004; Köhne et al. 2005; Javaux and Vanclooster 2006). Using a suction plate with a lower boundary pressure head of −500 cm we obtained a minimum pressure head between −149.9 and −235.4 cm. Thus in our study the range of water content and pressure head data was relatively wide for a column experiment. Large soil column experiments also face higher vertical heterogeneity compared to small cylinders (e.g. Inoue et al. 2000; Abbasi et al. 2003b; Ritter et al. 2004; Schwärzel et al. 2006). This increases the number of parameters to be estimated (seven to twelve in the cited studies; in our case eight).

A main concern in inverse modelling is convergence of the optimization algorithm at a global minimum. Particularly when the number of optimized parameters is high, the risk of non-unique solutions increases. Some authors therefore used global optimizing approaches to better explore the parameter space and avoid convergence at a local minimum (e.g. Vrugt et al. 2008). Still when properly controlling fit statistics as well as varying starting values, also local search algorithms such as the Levenberg-Marquardt algorithm implemented in HYDURS 1D and used in this study can provide appropriate parameter estimates (Šimůnek and Hopmans 2002). We observed the highest standard error for *K*_*s*_, while it was substantially lower for the retention parameters. Other studies with similar experimental setup did not report this indicator of parameter quality for comparison. Still overall R^2^ and SSQ values of the minimized objective function were in the same range as those reported by Abbasi et al. (2003b) who optimized up to 12 parameters. Other studies with different setup also reported highest uncertainty for K_s_ among optimized parameters (e.g. Vrugt et al. 2003). We suppose that this is related to the strongly non-linear response of K(h) to a slight departure from full saturation. Eching and Hopmans (1993) therefore considered *K*_*s*_ a mere fitting parameter without physical meaning in experiments that do not include full saturation. In a large soil column it is particularly difficult to establish saturation as an initial condition over the entire system. Concerning optimization quality we finally remark that our study was based on a randomized replicated design. Replication is rarely found in column experiments, probably due to cost constraints. In our case this setup allowed a proper statistical evaluation. Non-unique solutions would have increased the random error of parameter estimates due to convergence at different local minima. Statistical significance of treatment effects therefore provided an additional evidence for reliable optimization results.

Inverse estimation of hydraulic properties is based on the assumption that all relevant system properties are captured properly by the underlying hydraulic model. In our case this means that root induced changes were fully captured by changes in the optimized macroscopic Kosugi parameters (*θ*_*s*_, *r*_*m*_, *σ*, *K*_*s*_). Implicitly we claim that other potential root effects on flow properties such as wettability (e.g. Carminati 2012; Carminati and Vetterlein 2013) and pore connectivity (e.g. Whalley et al. 2005; Feeney et al. 2006) are of minor importance for the effective hydraulic behaviour of the system.

Concerning wettability, Carminati and Vetterlein (2013) indicate a mucilage affected zone around the root of 50 μm. Underlying a maximum root length density of 4.1 cm cm^−3^ measured in our experiment (mustard, 0–10 cm) as well as the corresponding average root diameter of 0.265 mm, about 1.0 % of total pore volume was directly affected by mucilage effects. Here it is assumed that the entire root length had a fully functional rhizosphere sheath. This small percentage of directly affected pore space therefore suggests that changes of contact angle by mucilage were insufficient to explain the differentiation in effective flow behaviour we observed and which resulted in significantly different hydraulic parameters. Consequently we are confident that mainly structural changes of material properties, i.e. frequency and distribution of pores as expressed in the macroscopic parameters, were the predominant reason for the differences we observed between planted and unplanted soil columns. Still we recognize that structural changes and mucilage are strongly related (e.g. Tisdall and Oades 1982). Furthermore the rhizosphere affected pore space might have been higher when considering additional root length (fine roots, hair roots) which was not captured by root washing and image analysis (Pierret et al. 2005). To further elucidate the role of mucilage on different scales, both theoretically and experimentally, root architecture models and drainage experiments with fluids of different contact angle with soil (e.g. water and ethanol; Jarvis et al. 2008) might be used.

The second implicit assumption concerns pore connectivity. In our study the related macroscopic parameter (tortuosity *l*) of the hydraulic conductivity function was fixed. In spite of this we claimed that the key pore parameters of interest in our study (*θ*_*s*_, *r*_*m*_, *σ*) could be correctly estimated. It is well known that plant roots enhance pore connectivity (e.g. Pagliai and De Nobili 1993; Whalley et al. 2005). Therefore distinct estimates of tortuosity between rooted and non-rooted treatments could be considered fundamental to capture root induced changes. The main reason for not including tortuosity in the optimization was to avoid an additional free parameters and thereby the risk of non-uniqueness of the optimized solution (Hopmans et al. 2002). Tortuosity is a poorly defined fitting parameter in macroscopic models of hydraulic conductivity (Vervoort and Cattle 2003). Thus it is difficult to define proper initial values and parameter constraints. Furthermore the parameter mostly affected by an inadequate tortuosity value is K_s_, while our study focused on root induced changes in PSD parameters (*θ*_*s*_, *r*_*m*_, *σ*). Fixing the tortuosity parameter in the hydraulic conductivity function, which is potentially treatment sensitive, implies that its effect on the observed water flow is incorporated into a correlated parameter. Elliot et al. (2008) among others showed that *K*_*s*_ is a function of pore connectivity and tortuosity. Thus mainly the optimized *K*_*s*_ values might have been biased by an effect that would have been otherwise attributed to distinct tortuosity. The higher values of *K*_*s*_ in the rooted columns indeed could indicate that upon optimization the potentially enhanced pore connectivity was compensated by increasing *K*_*s*_ to properly meet the observed water flow. We recognize that parameter correlation in macroscopic hydraulic property models is a general problem for optimization (Pollacco et al. 2013). Still we consider that reduction of free parameters in the hydraulic conductivity function was more advantageous for properly estimating the water retention parameters of interest here, than obtaining a physically unclear approximation for the root effect on pore connectivity.

In this context we notice that in spite of a good statistical fit between measured and simulated outflow as shown in Table 3 it cannot be excluded that part of the physics of the system (e.g. tortuosity, contact angle) are not captured entirely. In this context the limits of macroscopic models have to be recognized. In order to account for root induced changes in pore geometry (e.g. Feeney et al. 2006) pore network models (e.g. Vogel 2000, Holtham et al. 2007) would be more appropriate. Also multi-modal models with distinct tortuosity of each domain (e.g. Dexter and Richard 2009) might be considered. Still parameterization of such models is challenging (Šimůnek et al. 2003).

### Root influence on pore evolution

Modelling root driven pore dynamics first requires understanding the main changes in the PSD of rooted soil. Analysis of variance of the inversely determined hydraulic parameters revealed that there were two processes involved in the temporal change of hydraulic properties between the initial and final experiment, i.e. soil settlement and root influences. Soil settlement was expressed by the reduction of total porosity (pore closure), drift towards smaller pore classes and heterogeneization of the pore system (larger standard deviation of the PSD). These changes were more pronounced in the lower layer of the soil columns than in the upper layer. Rühle et al. (2013) recently observed a similar temporal trend towards a more heterogeneous pore system and non-uniform hydraulic behaviour during long-term soil column experiments.

Importantly we found significant differences in pore evolution between the non-planted and planted soil columns. This provided clear evidence of short term influences of roots on the PSD which have to be considered to describe the hydraulic behaviour of soil. Roots stabilized porosity, reducing pore loss (higher θ_s_) due to soil settlement and enhanced heterogeneity of the pore system (increase in the pore radius standard deviations). Horn et al. (1994) and Dexter and Richard (2009) remarked that heterogenization of the pore system is an important indicator of structural porosity and results from the formation of both coarse intra-aggregate and fine inter-aggregate pores upon soil structuring. On the contrary non-planted columns showed structure degradation expressed by a narrowing of the PSD. Bodner et al. (2013) reported similar dynamics from a field study with the same treatments (bare soil, mustard, rye) where planted plots also showed a significantly higher σ.

Also an increase in K_s_ in the rooted columns compared to the non-planted treatment was found. As shown by Hayashi et al. (2006), *K*_*s*_ would be related to both *r*_*m*_ as well as *σ*. The main reason for this is the strong influence of highly conductive and less tortuous macropores. The higher *σ* found in the planted columns implied that there was also an increase in the volume of high transmission macropores which strongly influenced *K*_*s*_ (*cf*. Table 6). However the main volumetric change related to the root induced increase in *σ* was found in the micropore volume.

Having identified significant root induced changes in macroscopic parameters (*θ*_*s*_, *σ*, *K*_*s*_), we could then try to identify relevant processes leading to the distinct changes in different pore classes. According to Watt et al. (2006) the volume of pores with a radius between 15 and 1,000 μm is the main space of root growth. Zobel (2008) indicated that fine roots, building up to 95 % of total root length, have been measured to a diameter as small as 60 μm. Thus we can assume that roots penetrate pores with a radius down to 30 μm, i.e. mainly the macropore range. Smaller pores can be accessed by root hairs (diameter around 10 μm; Jungk 2001) and mycorrhizal hyphae (diameter between 2 and 5 μm; Drew et al. 2003). Between 87.8 % (mustard) and 90.9 % (rye) of total root volume was within a radius range of <500 μm. Reduction in macro-and partially also mesoporosity (15 to 500 μm; 53 % of total decrease in pore volume in the rooted columns) of the rooted columns might be related to pore clogging by root in-growth into existing pore channels. The pore space directly occupied by roots however was relatively small during the 3.5 months growing time (0.13 to 0.02 % for mustard and rye respectively). Furthermore compared to the non planted columns higher reduction in pore volume of the rooted columns was found in mesopores and larger micropores between 2.5 and 15 μm. These pores are not directly accessible to root in-growth. Therefore pore clogging can only partially explain the observed pore dynamics in actively rooted soil. Pores <2.5 μm showed a substantial increase compared to the non-planted columns. Using the size classes given by Watt et al. (2006), roots thereby predominantly reduced the pore space between microaggregates (intra-aggregate space) while the pore space within these aggregates (inter-microaggregate porosity) increased. We suggest that capillary driven coalescence upon root water uptake (Leij et al. 2002) played a main role in the shift to higher microporosity. Also local compaction upon root penetration (Dexter 1987; Whalley et al. 2004; Aravena et al. 2011) might have increased micropore volume. Due to the dominance of fine axes in our model species however the extent of this process can be expected to be limited. The increase in pores <2.5 μm is of high functional importance due to their role in water storage. Recent results of Moradi et al. (2011) showed higher water content in the vicinity of plant roots. The authors suggested root mucilage as the main reason for this phenomenon, but also mention possible mechanical changes of soil pore configuration around roots. Although our method did not allow visualization of local phenomena, the bulk increase of storage pores in rooted soil is in agreement with this finding.

The higher value of *s* also resulted in an increase of larger macropores >500 μm in the rooted columns. Macropores are low in frequency but still they essentially contribute to overall porosity and hydraulic functioning of the soil. These pore class can be readily changed by localized effects in the vicinity of plant roots. The increase in macropores >500 μm in rooted columns points to such spatially heterogeneous effects. Carminati et al. (2009) described root shrinkage and air gap formation as a process of root induced macropore formation. Also fissures and cracks in the rhizosphere due to enhanced wetting and drying can act as newly formed macropores (Dexter 1987). The functional importance of these large pore classes is related the improved infiltrability and better soil aeration. The higher *K*_*s*_ in the rooted columns however can be a response to both, volumetric increase in macroporosity as well as higher pore connectivity.

Scaling between microscopic processes at the root-pore interface and changes in effective hydraulic behaviour is still a challenge. Effective parameters of the models used here were shown to be responsive to root effects. This provides quantitative evidence of the importance of roots for hydraulic processes. The changes in different pore classes point to distinct processes of root-soil structure interaction. However macroscopic models cannot extract the relative importance of single processes involved. This might be addressed more appropriately by spatially explicit root architecture models (e.g. Leitner et al. 2010) combined to pore network models (Holtham et al. 2007).

### Model framework for root induced pore evolution

When studying plant roots as drivers for the development of structural porosity, a dynamic description of PSD is required. Still in hydrological modelling, soil hydraulic properties are generally considered as fixed material property that does not change over time or under the influence of any structure forming agent (Šimůnek et al. 2003). There are only few quantitative approaches that have been suggested to describe pore evolution. Or et al. (2000) and Leij et al. (2002) have published a series of papers based on a convection–dispersion like equation to model temporal changes in hydraulic properties.

This model was shown to adequately describe all cases of pore drift towards a smaller *r*_*m*_ and a larger *σ*. However application of the model to the dynamics observed in our study revealed two problems. First, the model did not capture properly the dynamics in the non-planted columns where σ showed the reverse trend. Narrowing of the pore size distribution has been described for processes of structure degradation, such as aggregate disruption or soil compaction (e.g. Starsev and McNabb 2001; Hayashi et al. 2006). While Leij et al. (2002) successfully modelled post-tillage soil settlement; tillage itself would have induced a reverse process. Thus several naturally occurring pore dynamics would require a different modelling approach. Second, our results demonstrated that the linear relation between drift and dispersion via a constant dispersivity could not capture appropriately the changes in PSD with increasing rooting density. This might be related to an inadequate drift function. The sharp drift between a non-rooted and rooted soil suggests that other mechanisms than rooting density (e.g. root exudation, non-detected fine roots or root hairs, compaction and particle rearrangement) have been involved. However our data showed that roots had a significant impact on σ while they did not significantly influence *r*_*m*_. In this case it was probably not adequate to functionally relate dispersion (change in *σ*) and drift. This indicates that there were qualitatively different processes involved or at least quantitative difference not captured by a linear relation. The pore evolution model properly characterizes a diffusion like process (shift from lower to higher entropy) equilibrating pore volume over radius with time. However this physical behaviour is questionable for the actively self-organizing biological root-microbe-soil system (Young and Crawford 2004) where energy driven processes lead to a higher order in soil structure.

The pore evolution model still provides a useful framework to study the nature of several root related processes. Ghezzehei and Or (2000) applied a physically based aggregate coalescence model to predict the drift and dispersion terms. Our results suggested an important role of root driven coalescence among other processes involved simultaneously over different pore ranges (clogging, compaction, stabilization). Radius dependent drift and dispersion might improve the capacity of the model to capture root effects. Still the complexity of the biologically active root-pore system makes the formulation of a comprehensive physically based model a challenge.

## Conclusions

We quantified the changes in the PSD of soil rooted by mustard and rye compared to a non rooted soil. Based on a column drainage experiment parameters of the lognormal Kosugi PSD model were determined by inverse optimization. Statistical indicators showed that the inverse procedure provided reliable estimates of the macroscopic pore parameters. Although roots can influence pore connectivity, tortuosity was fixed avoid optimizing an additional fitting parameter. From the inverse results, we concluded that retention parameters can be properly estimated by macroscopic models, while the strong influence of pore geometry on the hydraulic conductivity function requires other approaches such as pore network models. We demonstrated that besides general soil settlement over time, root effects were significantly influencing temporal dynamics of the PSD. Reduction in total porosity was significantly reduced by roots. Also heterogeneity of the pore space was increased in the rooted columns indicating an increase in structural porosity. The volume of large transmission macropores as well as fine storage pore was higher in the rooted compared to the non-planted columns. From the reduction in pore space accessible to roots we concluded that pore clogging was only of minor importance, while enhanced structuring by enmeshment and aggregate coalescence were suggested as dominant processes. Applying a pore evolution model we were able to simulate the drift to smaller pore radius classes and increased pore heterogeneity in the rooted columns, while structure degradation in the non rooted columns was not captured appropriately. When using a rooting density dependent drift function however, suggested that a diffusion like model of pore evolution is only partially appropriate to describe the complex processes of active structure formation by plant roots.

## Abbreviations

PSD: pore size distribution
*h*_*m*_: median pressure head
*r*_*m*_: median pore radius
*σ*: standard deviation of PSD
*θ*_*s*_: saturation water content
*θ*_*r*_: residual water content
*K*_*s*_: saturated hydraulic conductivity
*V*: drift term
*λ*: dispersivity

## References

[CR1] Abbasi F, Jacques D, Šimůnek J, Feyen J, van Genuchten MT (2003). Inverse estimation of the soil hydraulic and solute transport parameters from transient field experiments: heterogeneous soil. Trans ASAE.

[CR2] Abbasi F, Šimůnek J, Feyen J, van Genuchten MT, Shouse PJ (2003). Simultaneous inverse estimation of soil hydraulic and solute transport parameters from transient field experiments: homogeneous soil. Trans ASAE.

[CR3] Angers DA, Caron J (1998). Plant-induced changes in soil structure: processes and feedbacks. Plant-induced soil changes: processes and feedbacks.

[CR4] Aravena JE, Berli M, Ghezzehei TA, Tyler SW (2011). Effects of root-induced compaction on rhizosphere hydraulic properties - X-ray microtomography imaging and numerical simulations. Environ Sci Technol.

[CR5] Archer NAL, Quinton JN, Hess TM (2002). Below-ground relationships of soil texture, roots and hydraulic conductivity in two-phase mosaic vegetation in South-east Spain. J Arid Environ.

[CR6] Bengough AG(2012) Water dynamics of the root zone: Rhizosphere biophysics and its control on soil hydrology. Vadose Zone J 11. doi:10.2136/vzj2011.0111

[CR7] Bengough AG, McKenzie BM, Hallett PD, Valentine TA (2011). Root elongation, water stress, and mechanical impedance: a review of limiting stresses and beneficial root tip traits. J Exp Bot.

[CR8] Bodner G (2007) Cover cropping in water limited environments. A field and modelling study of hydrological and soil structural effects of cover crops and their impact on the water balance. Dissertation, University of Natural Resources and Life Sciences BOKU, Vienna

[CR9] Bodner G, Loiskandl W, Buchan G, Kaul HP (2008). Natural and management-induced dynamics of hydraulic conductivity along a cover-cropped field slope. Geoderma.

[CR10] Bodner G, Himmelbauer M, Loiskandl W, Kaul HP (2010). Improved evaluation of cover crop species by growth and root factors. Agron Sustain Dev.

[CR11] Bodner G, Scholl P, Loiskandl W, Kaul HP (2013). Environmental and management influences on temporal variability of near saturated soil hydraulic properties. Geoderma.

[CR12] Carminati A (2012) A model of root water uptake coupled with rhizosphere dynamics. Vadose Zone J 11:10.2136/vzj2011.0106

[CR13] Carminati A, Vetterlein D (2013). Plasticity of rhizosphere hydraulic properties as a key for efficient utilization of scarce resources. Ann Bot.

[CR14] Carminati A, Vetterlein D, Weller U, Vogel HJ, Oswald SE (2009). When roots lose contact. Vadose Zone J.

[CR15] Carminati A, Moradi AB, Vetterlein D, Vontobel P, Lehmann E, Weller U, Vogel HJ, Oswald SE (2010). Dynamics of soil water content in the rhizosphere. Plant Soil.

[CR16] Carof M, De Tourdonnet S, Coquet Y, Hallaire V, Roger-Estrade J (2007). Hydraulic conductivity and porosity under conventional and no-tillage and the effect of three species of cover crop in northern France. Soil Use Manag.

[CR17] Clark LJ, Whalley WR, Barraclough PB (2003). How do roots penetrate strong soil?. Plant Soil.

[CR18] Cresswell HP, Kirkegaard JA (1995). Subsoil amelioration by plant roots—the process and the evidence. Soil physics and hydrology. Aust J Soil Res.

[CR19] Cresswell HP, Smiles DE, Williams J (1992). Soil structure, soil hydraulic properties and the soil water balance. Aust J Soil Res.

[CR20] De Willigen P, Nielsen NE, Claassen N, Castrignanò AM, Smit AL, Bengough AG, Engels C, Van Noordwijk M, Pellerin S, der Geijn V (2000). Modelling water and nutrient uptake. Root methods, a handbook.

[CR21] Dexter AR (1987). Compression of soil around roots. Plant Soil.

[CR22] Dexter AR, Richard G (2009) The saturated hydraulic conductivity of soils with *n*-modal pore size distributions. Geoderma 154:76–85

[CR23] Disparte AA (1987) Effect of root mass density on infiltration among four Mediterranean dryland forages and two irrigated legumes. M.S. thesis. University of California, Riverside

[CR24] Drew EA, Murray RS, Smith SE, Jakobsen I (2003). Beyond the rhizosphere: growth and function of arbuscular mycorrhizal external hyphae in sands of varying pore sizes. Plant Soil.

[CR25] Durner W, Iden SC (2011). Extended multistep outflow method for the accurate determination of soil hydraulic properties near water saturation. Water Resour Res.

[CR26] Durner W, Schultze B, Zurmühl T, van Genuchten MT, Leij FJ, Wu L (1999). State-of-the-art in inverse modeling of inflow/outflow experiments. Proceedings of the international workshop on characterization and measurement of the hydraulic properties of unsaturated porous media.

[CR27] Eching SO, Hopmans JW (1993). Optimization of hydraulic functions from transient outflow and soil water pressure data. Soil Sci Soc Am J.

[CR28] Elliot TR, Reynolds WD, Heck RJ (2008) Measuring pore contribution to Ksat flow using μCT scanning. Geophys Res Abstr 10:EGU2008-A-00809

[CR29] Feeney DS, Crawford JW, Daniell T, Hallett PD, Nunan N, Ritz K, Rivers M, Young IM (2006). Three-dimensional micro organization of the soil–root–microbe system. Microb Ecol.

[CR30] Fila G, Bellocchi G, Acutis M, Donatelli M (2003). IRENE: a software to evaluate model performance. Eur J Agron.

[CR31] Gardner WR (1960). Dynamic aspects of water availability to plants. Soil Sci.

[CR32] Ghestem M, Sidle RC, Stokes A (2011). The influence of plant root systems on subsurface flow: implications for slope stability. Bioscience.

[CR33] Ghezzehei TA, Or D (2000). Dynamics of soil aggregate coalescence governed by capillary and tillage processes. Water Resour Res.

[CR34] Gish TJ, Jury WA (1983). Effect of plant roots and root channels on solute transport. Trans ASAE.

[CR35] Gregory PJ (2006). Plant roots: growth, activity and interactions with soil.

[CR36] Gregory PJ, Hutchinson DJ, Read DB, Jenneson PM, Gilboy WB, Morton EJ (2003). Non-invasive imaging of roots with high resolution X-Ray micro-tomography. Plant Soil.

[CR37] Gyssels G, Poesen J, Bochet E, Li Y (2005). Impact of plant roots on the resistance of soils to erosion by water: a review. Progr Phys Geogr.

[CR38] Hayashi Y, Kosugi K, Mizuyama T (2006). Changes in pore size distribution and hydraulic properties of forest soil resulting from structural development. J Hydrol.

[CR39] Himmelbauer ML, Loiskandl W, Kastanek F (2004). Estimating length, average diameter and surface area of roots using two different image analyses systems. Plant Soil.

[CR40] Holtham DA, Matthews GP, Scholefield DS (2007). Measurement and simulation of void structure and hydraulic changes caused by root-induced soil structuring under white clover compared to ryegrass. Geoderma.

[CR41] Hopmans JW, Šimůnek J, van Genuchten MT, Leij FJ, Wu L (1999). Review of inverse estimation of soil hydraulic properties. Proceedings of the international workshop, characterization and measurement of the hydraulic properties of unsaturated porous media.

[CR42] Hopmans JW, Šimůnek J, Romano N, Durner W, Dane JH, Topp GC (2002). Simultaneous determination of water transmission and retention properties—inverse methods. Methods of soil analysis, part 4, physical methods, Soil Sci Soc Am. Book Ser 5.

[CR43] Horn R, Smucker A (2005). Structure formation and its consequences for gas and water transport in unsaturated arable and forest soils. Soil Tillage Res.

[CR44] Horn R, Taubner H, Wuttke M, Baumgartl T (1994). Soil physical properties related to soil structure. Soil Tillage Res.

[CR45] Inoue M, Šimůnek J, Shiozawa S, Hopmans JW (2000). Simultaneous estimation of soil hydraulic and solute transport parameters from transient infiltration experiments. Adv Water Resour.

[CR46] Jarvis N, Etana A, Stagnitti F (2008). Water repellency, near-saturated infiltration and preferential solute transport in a macroporous clay soil. Geoderma.

[CR47] Javaux M, Vanclooster M (2006). Three-dimensional structure characterization and transient flow modelling of a variably saturated heterogeneous monolith. J Hydrol.

[CR48] Jungk A (2001). Root hairs and the acquisition of plant nutrients from soil. J Plant Nutr Soil Sci.

[CR49] Kirby JM, Bengough AG (2002). Influence of soil strength on root growth: experiments and analysis using a critical-state model. Eur J Soil Sci.

[CR50] Köhne JM, Mohanty BP, Simunek J (2005). Inverse dual-permeability modeling of preferential water flow in a soil column and implications for field-scale solute transport. Vadose Zone J.

[CR51] Kosugi K (1996). Lognormal distribution model for unsaturated soil hydraulic properties. Water Resour Res.

[CR52] Kosugi K, Inoue M (2002). Estimation of hydraulic properties of vertically heterogeneous forest soil from transient matric pressure data. Water Resour Res.

[CR53] Kutschera L, Lichtenegger E, Sobotik M (2009). Wurzelatlas der Kulturpflanzen gemäßigter Gebiete mit Arten des Feldgemüsebaues.

[CR54] Leij FJ, Ghezzehei TA, Or D (2002). Modeling the dynamics of the soil pore-size distribution. Soil Tillage Res.

[CR55] Leitner D, Klepsch S, Bodner G, Schnepf S (2010) A dynamic root system growth model based on L-Systems. Tropisms and coupling to nutrient uptake from soil. Plant Soil. doi:10.1007/s11104-010-0284-7

[CR56] Littell RC, Henry PR, Ammerman CB (1998). Statistical analysis of repeated measures data using SAS procedures. J Anim Sci.

[CR57] Liu A, Ma BL, Bomke AA (2005) Effects of cover crops on soil aggregate stability, total organic carbon, and polysaccharides. Soil Sci Soc Am J 69:2041–2048

[CR58] Logsdon SD (2013) Root effects on soil properties and processes: synthesis and future research needs. In: Timlin T, Ahuja LR (eds) Enhancing understanding and quantification of soil–root growth interactions. Adv Agric Syst Model 4. doi:10.2134/advagricsystmodel4.c8

[CR59] Meek BD, Detar WR, Rolph D, Rechel ER, Carter LM (1990). Infiltration rate as affected by an alfalfa and no-till cotton cropping system. Soil Sci Soc Am J.

[CR60] Mitchell AR, Ellsworth TR, Meek BD (1995). Effect of root systems on preferential flow in swelling soil. Commun Soil Sci Plant Anal.

[CR61] Mooney SJ, Pridmore TP, Helliwell J, Bennett MJ (2012). Developing X-ray computed tomography to non-invasively image 3-D root systems architecture in soil. Plant Soil.

[CR62] Moradi AB, Carminati A, Vetterlein D, Vontobel P, Lehmann E, Weller U, Hopmans JW, Vogel HJ, Oswald SE (2011). Three-dimensional visualization and quantification of water content in the rhizosphere. New Phytol.

[CR63] Morgan RPC, Quinton JN, Edwards J (1995) Vegetation strategies for combating desertification. MEDALUS II Project 3 Managing Desertification. Contract EV5V-CT92-0165. Final Report

[CR64] Murphy B, Koen T, Jones B, Huxedurp L (1993). Temporal variation of hydraulic properties for some soils with fragile structure. Aust J Soil Res.

[CR65] Or D, Leij F, Snyder V, Ghezzehei TA (2000). Stochastic model for post tillage soil pore space evolution. Water Resour Res.

[CR66] Pagliai M, De Nobili M (1993). Relationships between soil porosity, root development and soil enzyme activity in cultivated soils. Geoderma.

[CR67] Piepho HP, Büchse A, Richter C (2004). A mixed modelling approach for randomized experiments with repeated measures. J Agron Crop Sci.

[CR68] Pierret A, Moran CJ, Doussan C (2005). Conventional detection methodology is limiting our ability to understand the roles and functions of fine roots. New Phytol.

[CR69] Pollacco JA, Nasta P, Soria-Ugalde JM, Angulo-Jaramillo R, Lassabatere L, Mohanty BP, Romano N (2013). Reduction of feasible parameter space of the inverted soil hydraulic parameters sets for Kosugi model. Soil Sci.

[CR70] Ritter A, Hupet F, Muñoz-Carpena R, Lambot S, Vanclooster M (2003). Using inverse methods for estimating soil hydraulic properties from field data as an alternative to direct methods. Agric Water Manag.

[CR71] Ritter A, Muñoz-Carpena R, Regalado CM, Vanclooster M, Lambot S (2004). Analysis of alternative measurement strategies for the inverse optimization of the hydraulic properties of a volcanic soil. J Hydrol.

[CR72] Rühle FA, Klier C, Stumpp C (2013). Changes in water flow and solute transport pathways during long-term column experiments. Vadose Zone J.

[CR73] Scanlan CA (2009) Processes and effects of root-induced changes to soil hydraulic properties. Dissertation. University of Western Australia

[CR74] Scanlan CA, Hinz C (2010). Using radius frequency distribution functions as a metric for quantifying root systems. Plant Soil.

[CR75] Schaap MG, Leij FJ, van Genuchten MT (2001). ROSETTA: a computer program for estimating soil hydraulic parameters with hierarchical pedotransfer functions. J Hydrol.

[CR76] Schwärzel K, Šimůnek J, Stoffregen H, Wessolek G, Van Genuchten MT (2006). Estimation of the unsaturated hydraulic conductivity of peat soils. Vadose Zone J.

[CR77] Schwärzel K, Carrick S, Wahren A, Feger KH, Bodner G, Buchan G (2011). Soil hydraulic properties of recently tilled soil under cropping rotation compared with two-year pasture. Vadose Zone J.

[CR78] Schwen A, Bodner G, Scholl P, Buchan GD, Loiskandl W (2011). Temporal dynamics of soil hydraulic properties and the water-conducting porosity under different tillage. Soil Tillage Res.

[CR79] Šimůnek J, Hopmans JW, Dane JH, Topp GC (2002). Parameter optimization and non linear fitting. Methods of soil analysis. Part 4. Physical methods. SSSA Book Ser 5.

[CR80] Šimůnek J, Angulo-Jaramillo R, Schaap MG, Vandervaere JP, van Genuchten MT (1998). Using an inverse method to estimate the hydraulic properties of crusted soils from tension-disc infiltrometer data. Geoderma.

[CR81] Šimůnek J, Jarvis NJ, Van Genuchten MT, Gärdenäs A (2003). Review and comparison of models for describing non-equilibrium and preferential flow and transport in the vadose zone. J Hydrol.

[CR82] Šimůnek J, Šejna M, Saito H, Sakai M, van Genuchten MT (2013). The Hydrus-1D software package for simulating the movement of water, heat, and multiple solutes in variably saturated media, version 4.16, HYDRUS software series 3, Department of Environmental Sciences.

[CR83] SSSA (2013) Soil Science Society of America—Glossary of soil science terms. Pore size classification. www.soils.org/files/publications/soils-glossary/table2.pdf

[CR84] Starsev AD, McNabb DH (2001). Skidder traffic effects on water retention, pore-size distribution, and van Genuchten parameters of boreal forest soils. Soil Sci Soc Am J.

[CR85] Suwardji P, Eberbach P (1998). Seasonal changes of physical properties of an oxicpaleustalf (red kandosol) after 16 years of direct drilling or conventional cultivation. Soil Tillage Res.

[CR86] Tisdall JM, Oades JM (1982) Organic matter and water-stable aggregates in soils. J Soil Sci 62:141–163

[CR87] Van Genuchten MT, Leij FJ, Yates SR (1991). The RETC code for quantifying the hydraulic functions of unsaturated soils, Version 1.0. EPA Report 600/2-91/065, U.S. Salinity Laboratory.

[CR88] Vervoort RW, Cattle SR (2003) Linking hydraulic conductivity and tortuosity parameters to pore space geometry and pore-size distribution. J Hydrol 272:36–49

[CR89] Vidal M, López A (2005). Cover crops and organic amendments to prevent nitrate contamination under a wet climate. Agron Sustain Dev.

[CR90] Vogel HJ (2000) A numerical experiment on pore size, pore connectivity, water retention, permeability, and solute transport using network models. Eur J Soil Sci 51:99–105

[CR91] Vrugt JA, Bouten W, Grupta HV, Hopmans JW (2003) Toward improved identifiability of soil hydraulic parameters. Vadose Zone J 2:98–113

[CR92] Vrugt JA, Stauffer PH, Wöhling T, Robinson BA, Vesselinov VV (2008). Inverse modeling of subsurface flow and transport properties: a review with new developments. Vadose Zone J.

[CR93] Watt M, Silk WK, Passioura JB (2006). Rates of root and organism growth, soil conditions, and temporal and spatial development of the rhizosphere. Ann Bot.

[CR94] Whalley WR, Leeds-Harrison PB, Leech PK, Riseley B, Bird NRA (2004). The hydraulic properties of soil at root-soil interface. Soil Sci.

[CR95] Whalley WR, Riseley B, Leeds-Harrison PB, Bird NRA, Leech PK, Adderley WP (2005). Structural differences between bulk and rhizosphere soil. Eur J Soil Sci.

[CR96] Williams SM, Weil RR (2004). Crop cover roots may alleviate of soil compaction effects on soybean crop. Soil Sci Soc Am J.

[CR97] Wuest SB (2001). Soil biopore estimation: effects of tillage, nitrogen, and photographic resolution. Soil Tillage Res.

[CR98] Yang H, Rahardjo H, Wibawa B, Leong EC (2004). A soil column apparatus for laboratory infiltration study. Geotech Test J.

[CR99] Young IM (1998). Biopysical interactions at the root-soil interface: a review. J Agric Sci (Camb).

[CR100] Young IM, Crawford JW (2004). Interactions and self-organization in the soil-microbe complex. Science.

[CR101] Yunusa IAM, Newton PJ (2003). Plants for amelioration of subsoil constraints and hydrological control: the primer-plant concept. Plant Soil.

[CR102] Zobel RW (2008). Hardware and software efficacy in assessment of fine root diameter distributions. Comput Electron Agric.

[CR103] Zuazo VHD, Pleguezuelo CRR (2008). Soil-erosion and runoff prevention by plant covers. A review. Agron Sustain Dev.

